# Structure to Property: Chemical Element Embeddings for Predicting Electronic Properties of Crystals

**DOI:** 10.1021/acs.jcim.3c01990

**Published:** 2024-07-15

**Authors:** Shokirbek Shermukhamedov, Dilorom Mamurjonova, Thana Maihom, Michael Probst

**Affiliations:** † Institute of Ion Physics and Applied Physics, University of Innsbruck, 6020 Innsbruck, Austria; ‡ Department of Inorganic Chemistry, Tashkent Chemical Technological Institute, 100011 Tashkent, Uzbekistan; § School of Molecular Science and Engineering, Vidyasirimedhi Institute of Science and Technology, 21201 Rayong, Thailand; ∥ Division of Chemistry, Department of Physical and Material Sciences, Faculty of Liberal Arts and Science, Kasetsart University, Kamphaeng Saen Campus, 73140 Nakhon Pathom, Thailand

## Abstract

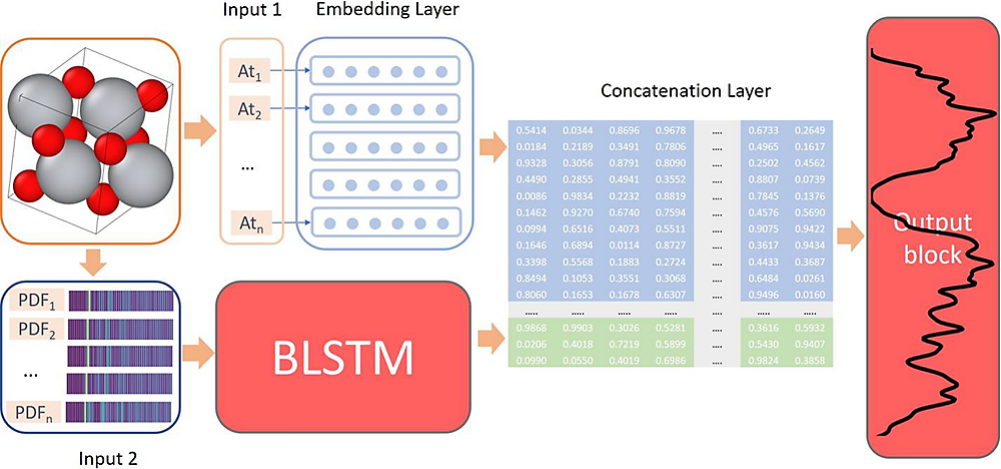

We present a new general-purpose machine learning model that is able to predict a variety of crystal properties, including Fermi level energy and band gap, as well as spectral ones such as electronic densities of states. The model is based on atomic representations that enable it to effectively capture complex information about each atom and its surrounding environment in a crystal. The accuracy achieved for band gaps exceeds results previously published. By design, our model is not restricted to the electronic properties discussed here but can be extended to fit diverse chemical descriptors. Its advantages are (a) its low computational requirements, making it an efficient tool for high-throughput screening of materials; and (b) the simplicity and flexibility of its architecture, facilitating implementation and interpretation, especially for researchers in the field of computational chemistry.

## 1 Introduction

Machine learning (ML) methods are widely used in computational chemistry to forecast experimental data and electronic properties across various chemical systems, including batteries,^1,2^ supercapacitors,^3^ thermoelectric^4^ and photoelectric^5^ compounds, and are used in catalysis^6^ and drug design^7^ and to explore the properties of materials^8^ and their surfaces under extreme conditions.^9^ The key point of ML methods is structure representation, or the encoding of the atomic environment of a given compound or chemical system. Numerous ML techniques have been proposed to determine the relationship between composition, structure and properties in material design. The variety of current approaches includes descriptor-based representations, feature engineering, and deep learning techniques.^10^ Among these, graph neural networks (GNNs)^11^ and high-dimensional neural networks (HDNNs)^12^ emerge as the two most commonly used approaches for structure representation. We provide a brief comparison between these two methods.

GNNs transform molecular graphs into node and edge representations, offering state-of-the-art performance for tasks related to materials.^11,13−19^ HDNNs, on the other hand, convert Cartesian coordinates of atoms into environmental representations using predefined or learnable descriptors.^20−26^ Both GNN and HDNN models have shown significant success in quantum chemical property calculations.^25,27^ Each approach has its own advantages and disadvantages, and computational costs remain high, with a significant impact on the type of training data set.

Electronic density of states (eDOS) is a key quantity that describes the electronic properties of materials. Accurate prediction of eDOS is essential for understanding and designing new materials with desired electronic properties. The calculation of DOS with quantum chemical methods is computationally very expensive, thereby impeding high-throughput screening of compounds. Furthermore, computing DOS for compounds comprising thousands of atoms is a daunting task in quantum chemistry due to the scaling of the more accurate methods. Hence, the application of ML techniques offers significant advantages in addressing such computational challenges. Recently, GNNs have been used to predict the spectral properties of crystal structures.^14,28−30^ Fung et al. used a GNN model to predict eDOSs of alloy surfaces and bulk crystal materials.^28^ To calculate the phonon density of states (phDOS) and eDOS of crystalline materials, Kong et al. adapted graph attention networks (GATs) GNN and proposed the Mat2Spec model.^14^ Its predictions of ground-truth eDOS gave a mean absolute error (MAE) of 3.64 states/eV, the best result among various tested models, including GATGNN and Euclidian Neural Network models (E3NN). Euclidian Neural Networks have also been applied to predict phDOS based only on crystal structure.^29^ The atomic positional embedding-based transformer (APET) approach was employed to predict eDOS, yielding an MAE score of 2.687^31^ for the extended Materials Project database. APET uses atomic positions in its embedding to capture detailed structural information in a crystal. Bang et al. used a crystal graph convolutional neural networks (CGCNN) to predict the eDOS of metal nanoparticles.^30^ The results are also compared with HDNN type SOAP- and MBTR-based models. The predictions of the latter were inferior to the GNN model. Mahmoud et al. proposed a sparse Gaussian process regression model based on SOAP descriptors to predict the eDOS of silicon structures.^32^ Despite the wide range of computational models, there is still room for more general and flexible models.

In this work, we present a HDNN type deep learning model designed for predicting the electronic properties of crystals, including eDOS. Our element embeddings model (EEM) integrates essential structural representations through pair distribution functions and atomic embedding representations as primary input parameters. This combination of inputs allows the model to understand the structural information on chemical compounds. To train the model, we used the extensive and diverse Materials Project (MP)^33^ structures database, which covers a wide range of crystal structures. The use of the MP data set enriches the training process, contributing to reliable and accurate predictions, thus increasing the applicability and relevance of the model in the field of computational materials science.

## 2 Method

### 2.1 Training Data Set

We performed calculations on two versions of the Materials Project^33^ data set. The first version, collected for training the Mat2Spec model (M2SM).^14^ This data set contains 38,688 structures with a unified energy range between *E*_min_ and *E*_max_. The second version, referred to as the MP2022 data set, was manually collected using the Materials Project API^33^ (API version 2022.7.8) and consists of a total of 126,334 crystals. This data set includes various quantum-chemically computed properties, such as formation energy, band gap, Fermi level energies, bulk moduli, and shear moduli. Moreover, eDOS patterns, computed under uniform computational conditions and employing a single pseudopotential, are accessible for 43,891 (35% of total structures) bulk structures within the MP2022 data set. Each structure includes eDOS spectra spanning various energy ranges defined by *E*_min_ and *E*_max_, with 2001 data points. Further details regarding this data set are provided in Section 1 of the Supporting Information.

### 2.2 Neural Network Architecture

**Figure 1:**
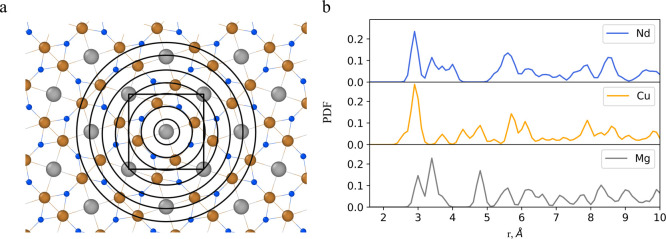
2D illustration of MgCu_2_Nd_2_ (a) and its PDFs (b). Blue spheres correspond to the neodymium (Nd) atoms, dark orange spheres represent Copper (Cu), and gray spheres denote magnesium (Mg) atoms. Same-element PDFs on the right side use the same colors. Black circles denote the boundaries between the histogram channels of the PDF. Square black frame indicates a unit cell. Atoms outside of it are periodic replicas.

In our approach, the pair distribution function (PDF) and element layers are employed as inputs to the neural network (NN). The PDF is defined as the probability of locating an atom at a given distance (r) from a reference atom.^34^ Being a basic quantity in statistical mechanics, it is widely used in materials science, chemistry, and condensed matter physics. Section 2 of the Supporting Information contains details regarding the PDF formalism. Specifically, this way of encoding neighborhood information has been successfully employed to predict the density of electronic states at Fermi energy.^35^Figure 1 provides a schematic illustration of atomic PDFs for a MgCu_2_Nd_2_ crystal. For instance, the first peak in a PDF corresponds to the most likely nearest-neighbor distances, reflecting the typical bond lengths present within the crystal structure (Figure 1b). To generate the training data, PDF calculations were performed with the ASE library^36^ with a cutoff radius of 10 Å.

The second input involves element embedding vectors. They encode essential information about the crystal in a more compact form, enabling machine learning models to effectively learn patterns and relationships. By encoding atomic information through the embedding layer, the model can discern subtle variations in the local environment of each atom and how these contribute to the overall structure of the material. In our workflow, all elements in the crystals are encoded as integers (nuclear charges can be used), forming an elemental vocabulary of size *V*_size_. Then, according to the number of atoms (*N*) in the crystals, the maximal length of input frames (*N*_max_) is defined. As a result, a single crystal structure is represented by two arrays: the 1 × *N*_max_ element vectors and the 100 × *N*_max_ structure matrix. If the specific crystal has fewer atoms, both arrays are padded with zeros to fit the NN input.

**Figure 2:**
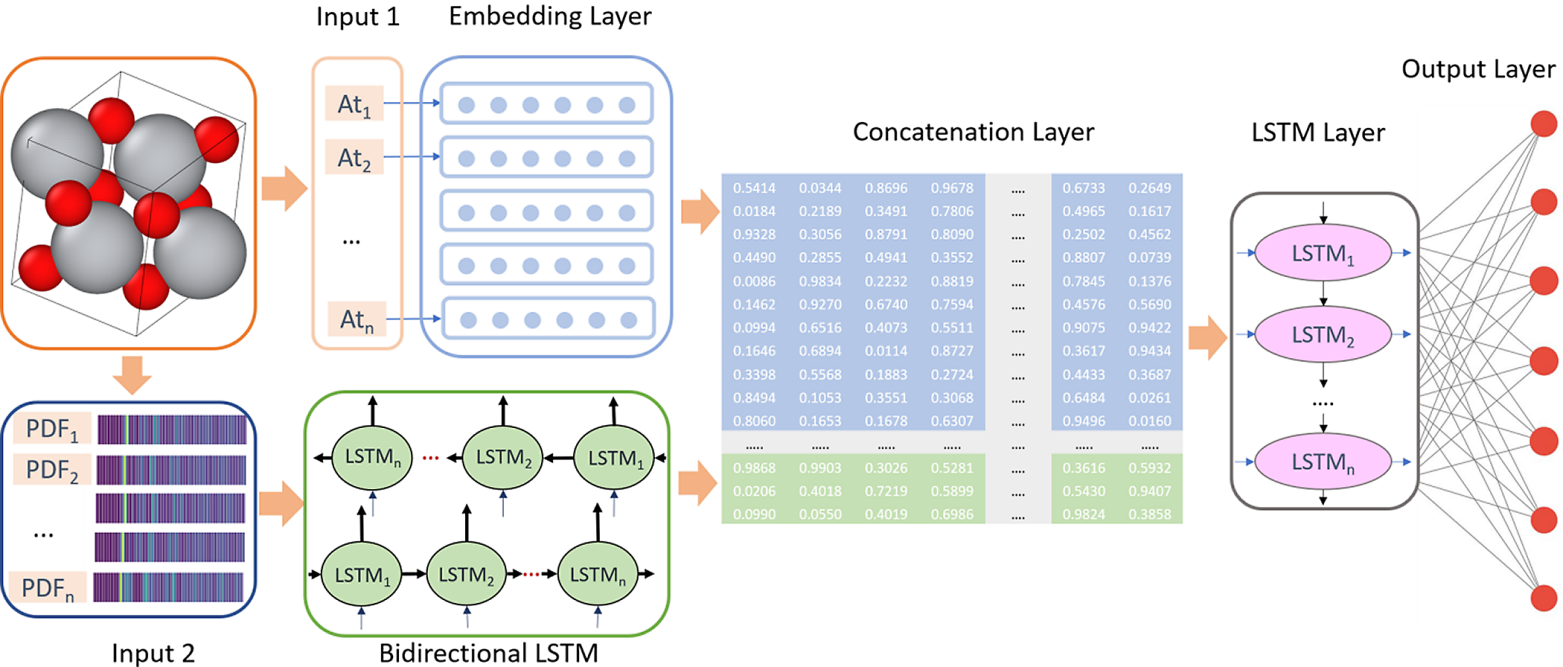
Model architecture. Two branches handle information about the crystal: First branch features an embedding layer that maps element indexes to embedding vectors. Second branch encodes pair distribution function (PDF) information using a bidirectional LSTM layer. Output from both branches is concatenated and passed through LSTM layer to predict output values.

Figure 2 shows a schematic representation of the model’s structure. The embedding layer, positioned atop the figure, maps element vectors to embedding vectors using their vocabulary indexes. These vectors are trainable parameters of the ML model. The bottom branch involves a bidirectional long–short-term memory (BLSTM) layer with 8-unit cells. BLSTM layers are a type of recurrent neural network architecture that can effectively capture dependencies in sequential data, such as the atomic arrangements represented by PDFs. Unlike traditional LSTM layers, which process sequential data in one direction, BLSTM layers process the input sequence in both forward and backward directions simultaneously. This bidirectional processing allows the model to consider short- and long-range information during training, thereby enhancing its ability to understand the complex relationships and patterns present in the PDFs. The output from both layers is concatenated in the subsequent step, and the resulting values are used with LSTM and output dense layers. In terms of model architecture, additional predictions of other quantities could be included alongside spectra, or scalar quantities can be fitted individually. Moreover, different types of neural network layers can be used instead of BLSTM and LSTM layers. Possible variations in the model architecture underscore the flexibility of our workflow and are presented in Section 3 of the Supporting Information.

### 2.3 Training Procedure

In the following sections, we will present the results of prediction models with an embedding size of 128. These models were trained using Keras.^37^ We split the data sets into three subsets: the training set, the validation set, and the test set, with an 80:10:10 ratio. Throughout the training process, we closely monitored the performance metrics, including the mean absolute error loss function and mean squared error (MSE). Additionally, we have used Mean Absolute Relative Percent Difference (MARPD) metrics to provide normalized accuracy measures. This approach enhances interpretability for those less familiar with eDOS measurements and facilitates accurate comparisons of model performance. This metric has been applied specifically to the benchmarks of the Materials Project.^38^ The MARPD value for the single spectra was computed using the following equation:
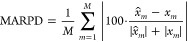
1where *m* represents the index of an eDOS value corresponding to *E*_*m*_, *M* is the total number of data points, *x*_*m*_ is the true value of the eDOS data point, and  is the model’s estimate of *x*_*m*_.

Also, we also used the *R*-squared (*R*^2^) metric that measures how well the model’s predictions match the comparable data, with a value of 1 indicating a perfect fit. Considering these metrics together provides a more comprehensive assessment of accuracy than any single metric can offer.

To train the models, we utilized the Adam optimizer with a learning rate of 0.001 while implementing a reduction in epochs after every 15 iterations. Furthermore, the batch size was fixed at 32, with the number of epochs spanning from 2 to 4 times the batch size to ensure comprehensive model convergence and performance evaluation.

## 3 Results

### 3.1 Mat2Spec Data Set

We initiated our calculation using the Mat2spec data set (M2SD)^14^ to validate the efficacy of our atomic workflow. The eDOS data in this data set spans energy windows ranging from −4 to 4 eV, with 128 data points, and the energy axis is adjusted so that the Fermi level energy is set to 0. We partitioned the reference data set into train, validation, and test sets following the original work.^14^ The training model consisted of 256 LSTM cells and 128 output neurons. Training was performed over 128 epochs, with a total training time of 25 min, averaging 11 s per epoch. After the training phase, we assessed the model’s performance using evaluation metrics.

**Figure 3:**
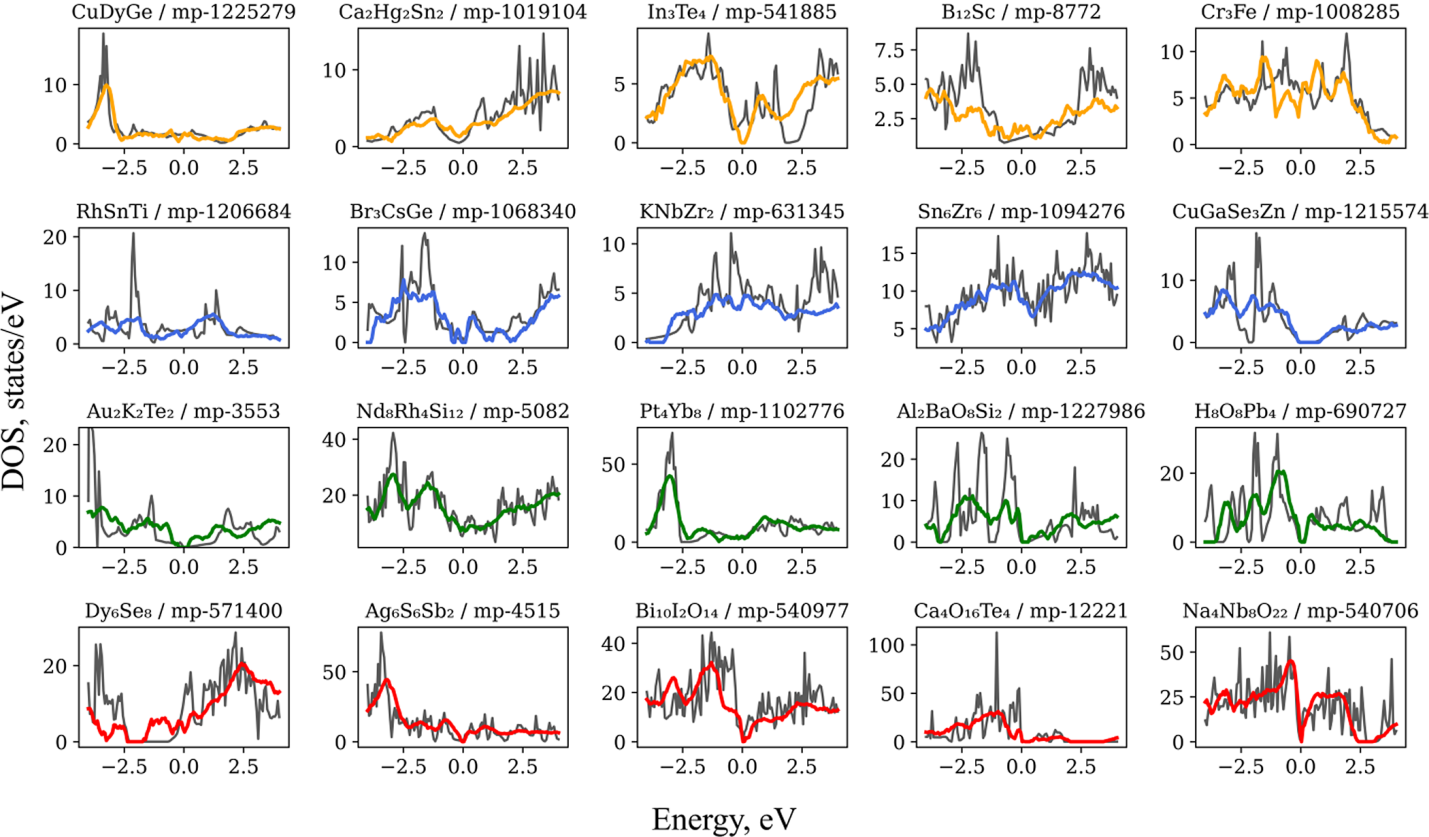
eDOS examples taken from the test set of the Mat2Spec data set (black) and the predicted spectra (colored). Examples are for five representative materials chosen from each quartile from low MAE (top) to high MAE (bottom) predictions, represented by colors transitioning from orange to red lines. Chemical sum formula and the ID number of each material are shown above each subplot.

Table 1 summarizes the results of various models trained on the M2SD using MAE loss. Figure 3 shows the predicted eDOS spectra generated by our workflow, grouped into quartiles based on their MAE values. The plots are arranged from the top (lowest MAE) to bottom (highest MAE). Although our results reasonably approximate the general behavior of eDOS spectra, like the M2SM, the EEM spectra struggle to accurately reproduce the narrow peaks and deep holes observed in the reference data set. The EE model achieved an MAE (states/eV) value of 3.91 and an MSE (states^2^/eV^2^) value of 86.03. The original M2SM was trained and executed using various combinations of data scaling and training loss functions, creating an ensemble of separately optimized models. Among these, the most successful combinations use SumNorm scaling and KL loss, achieving an MAE of 3.8 and a MSE of 74.5, while standard scaling with MAE loss resulted in an MSE value of 80.4 and an MAE of 3.64.

**Table 1:** Results of eDOS Prediction on the M2SD Test Set, with Mat2Spec, GATGNN, and E3NN Values Obtained from Kong et al.^14^ and APET Results Gained from Cui et al.^31^^a^

model	MAE	MSE	MARPD
Mat2Spec	3.64	80.4	28.28
GATGNN	4.89	120.9	
E3NN	5.24	105.1	
APET	3.72	80.84	
EEM (this work)	3.91	86.03	26.81
base (this work)	4.78	112.67	32.09

a Construction of our EEM is described in Section 3.2, and our Base model is described at the end of Section 3.1.

**Figure 4:**
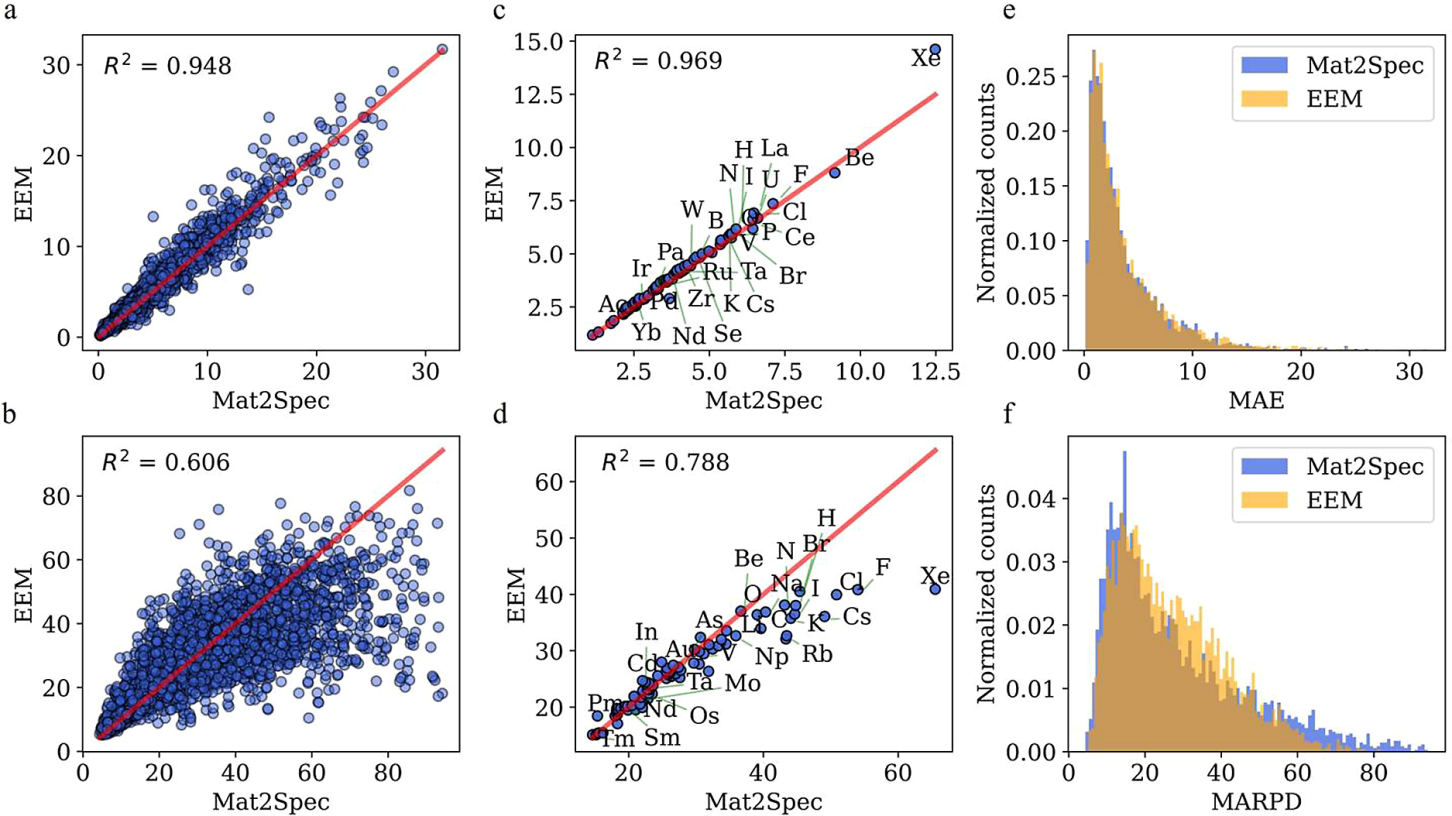
Performance evaluation and error distribution of M2SM and EEM models across crystals and elemental compositions. (a) Correlation plot of MAE values for both models for each crystal. (b) Similar correlation plot for MARPD values. Average MAE (c) and MARPD values (d) across different elements included in the crystals. Distribution plot of MAE values (e) and MARPD values (f) for both models. Red line corresponds to the perfect linear correlation (*R*^2^ = 1) between the Mat2Spec predictions and the EEM values.

Comparing performance metrics between our model and M2SM reveals noteworthy insights. While our model’s MAE and MSE values are slightly higher than those of M2SM, the MARPD values are closer. Specifically, M2SM has an average MARPD of 28.28, whereas our model’s MARPD is 26.81. To compare our workflow with M2SM in more detail, we performed a comprehensive analysis of both outputs. In this analysis, the M2SM predictions are treated as benchmark data. Figure 4 presents several correlation plots and histograms of the MAE (top) and MARPD (bottom) metrics. In the correlation plots, the *x*-axis represents the M2SM values, while the *y*-axis represents the values from EEM.

Figure 4a shows the correlation plot of MAE values predicted by M2SM and EEM for each crystal, with an *R*^2^ value of 0.948. Figure 4b presents a similar plot for the MARPD metric. Qualitatively, a lower MARPD value indicates better performance. With an *R*^2^ of 0.606, our models predict more crystals with lower MARPD values. We also investigated prediction quality based on the elemental composition of the crystals. Figure 4c,d show the average MAE and MARPD values, respectively, for the elements present in test set crystals. In this case, the MAE *R*^2^ is 0.969, compared to 0.788 for MARPD. The EEM predictions exhibit significantly lower values compared to M2SM. Finally, Figure 4e,f show the distribution of crystal values for each model, confirming previous trends. EEM predictions are more accurate where M2SM has larger errors, though EEM produces higher errors in the 20–40 MARPD range. Overall, both models exhibit their strengths and weaknesses, and this comparison confirms the comparability of the two models.

Furthermore, it is noteworthy that our approach outperforms other GNN models examined in Kong et al.,^14^ particularly E3NN and GATGNN. Considering the complexity of GNNs in general and the fact that M2SM is specifically designed to predict spectra, we can confidently assert the superiority of our EEM architecture.

The robustness of the predictive performance of our workflow can be attributed to the inherent correlations captured by element embeddings. To verify this, we performed additional calculations using a simple dense layer over element types instead of embeddings (Base model). The resulting MAE of 4.78 and MSE of 112.67 were inferior to the original model with embedding layers. For further illustrative purposes, examples of the resulting eDOS are presented in Figure S4 in the Supporting Information.

Despite the promising predictive powers of our model, there is potential for further refinement to capture the fine details present in eDOS patterns. In addition, one can aim to improve the predictions for other electronic quantities within the MP data set.

### 3.2 MP2022 Data Set

Leveraging the extensive MP2022 data set poses unique challenges compared to the standardized M2SD, necessitating additional inputs and outputs. Quantum chemical packages compute spectra within predefined energy ranges, with eDOS energies typically adjusted by shifting the energy axis to the Fermi level (as in Mat2Spec) and normalizing the eDOS values to the number of electrons (*N*_e_) in the compound, represented as .

**Figure 5:**
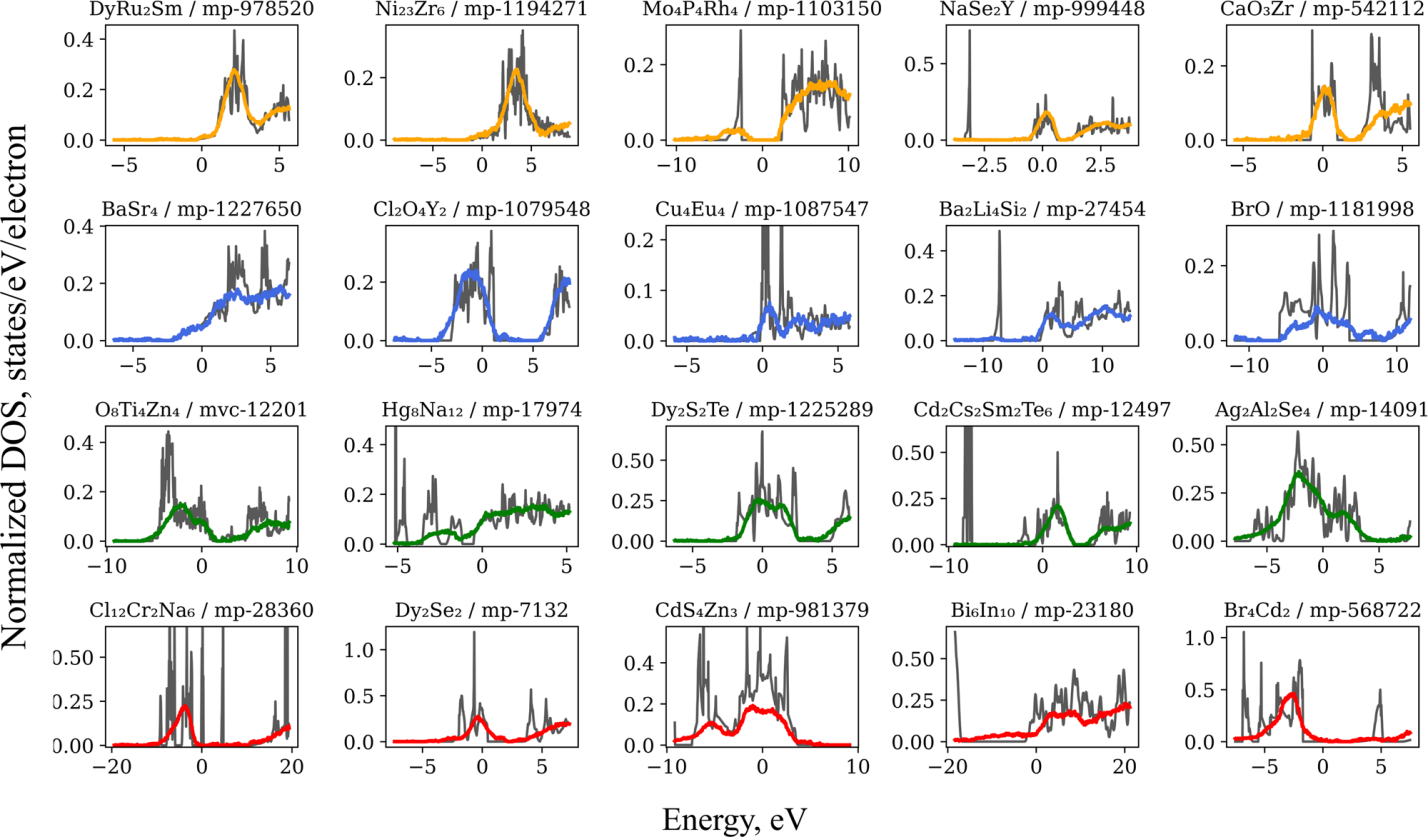
eDOS examples taken from the test set of the MP2022 data set (normalized to the number of electrons, black), and predicted spectra (colored). Examples consist of five representative materials chosen from each quartile of EEM predictions, arranged from low MAE (top) to high MAE (bottom), represented by colors transitioning from orange to red lines. Chemical sum formula and ID number of each material are shown above each subplot.

In our approach, we trained two models to predict absolute eDOS values: one using standard MinMax normalization and the other using normalized values obtained with the provided equation (NeN model). However, we did not shift them to the Fermi level energy. Instead, we selected 256 data points from both sides of the eDOS spectrum centered around zero values of energy and attempted to predict spectra, Fermi level energies, and band gap values. These adjustments required modifications to the model architecture, including an additional input branch with dense layers to account for the energy vectors of the spectra. The output from this branch was concatenated with the outputs from the BLSTM and Embeddings branches. The resulting layer was then passed through the LSTM and dense layers to predict the output quantities. The compiled models were trained for 256 epochs. The predicted spectra obtained by NeN model are presented in Figure 5.

Predictions of absolute eDOS values achieved an MAE value of 2.49 and a MARPD value of 42.52, with examples of eDOS spectra provided in Figure S5 of the Supporting Information. This MAE value outperforms the one obtained from other embeddings based APET model (2.687).^31^ Additionally, the MAE for the Fermi level energy was 0.232 eV, while the band gap value showed an MAE of 0.199 eV, demonstrating the accuracy of the predictive model.

**Figure 6:**
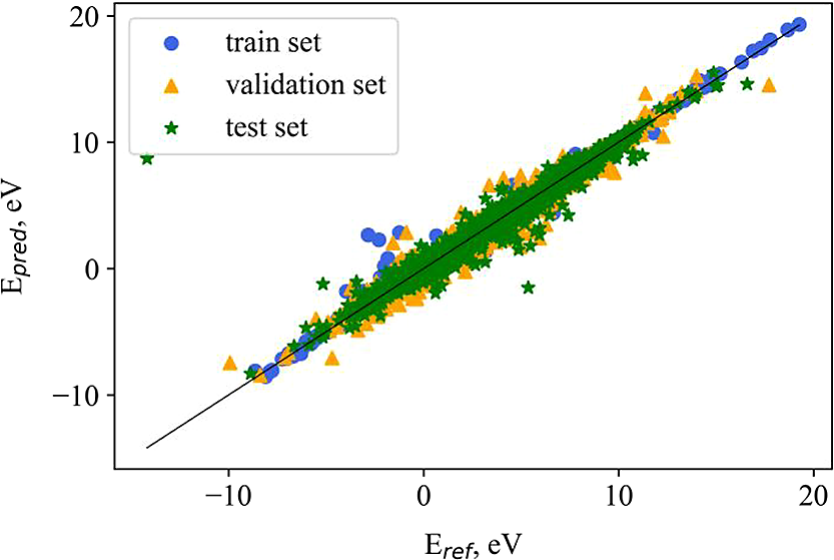
Correlation between predicted and reference fermi level energies from the MP2022 data set.

An analysis of N_e_ normalized eDOS predictions revealed a MARPD value of 42.92, with the Fermi level energy MAE reported as 0.230 eV. This value exceeds those predicted by CGCNN model, which had a value of 0.363 eV.^18^ The correlation between reference and predicted Fermi level energies, divided into training, validation, and test sets, is presented in Figure 6. The band gap values of crystals in the test set had an MAE value of 0.196 eV, which is superior to the best results reported from GNN models (0.204 eV).^19^ It should be noted that we used only a subset of the extensive MP data set, similar to the reference GNN model.

In comparing two trained models employing distinct normalization techniques, it is noteworthy that despite minor variations in comparable values such as MARPD, Fermi levels and band gaps, the NeN model exhibited qualitative advantages. Figure S6 compares examples of eDOSs obtained from both models. Further quantitative and qualitative comparisons, including those with the M2SD trained model, will be conducted in the next section.

## 4 Discussion

Starting from the numerical and visual comparisons presented up to here, a deeper analysis can lead to more insights. By examining the relationship between MARPD and the elemental composition of crystal, we aim to identify further nuances among the trained models. This analysis extends the methodology used in Figure 3c,d and aims to explain why crystals with certain elements consistently show high errors across all models.

**Figure 7:**
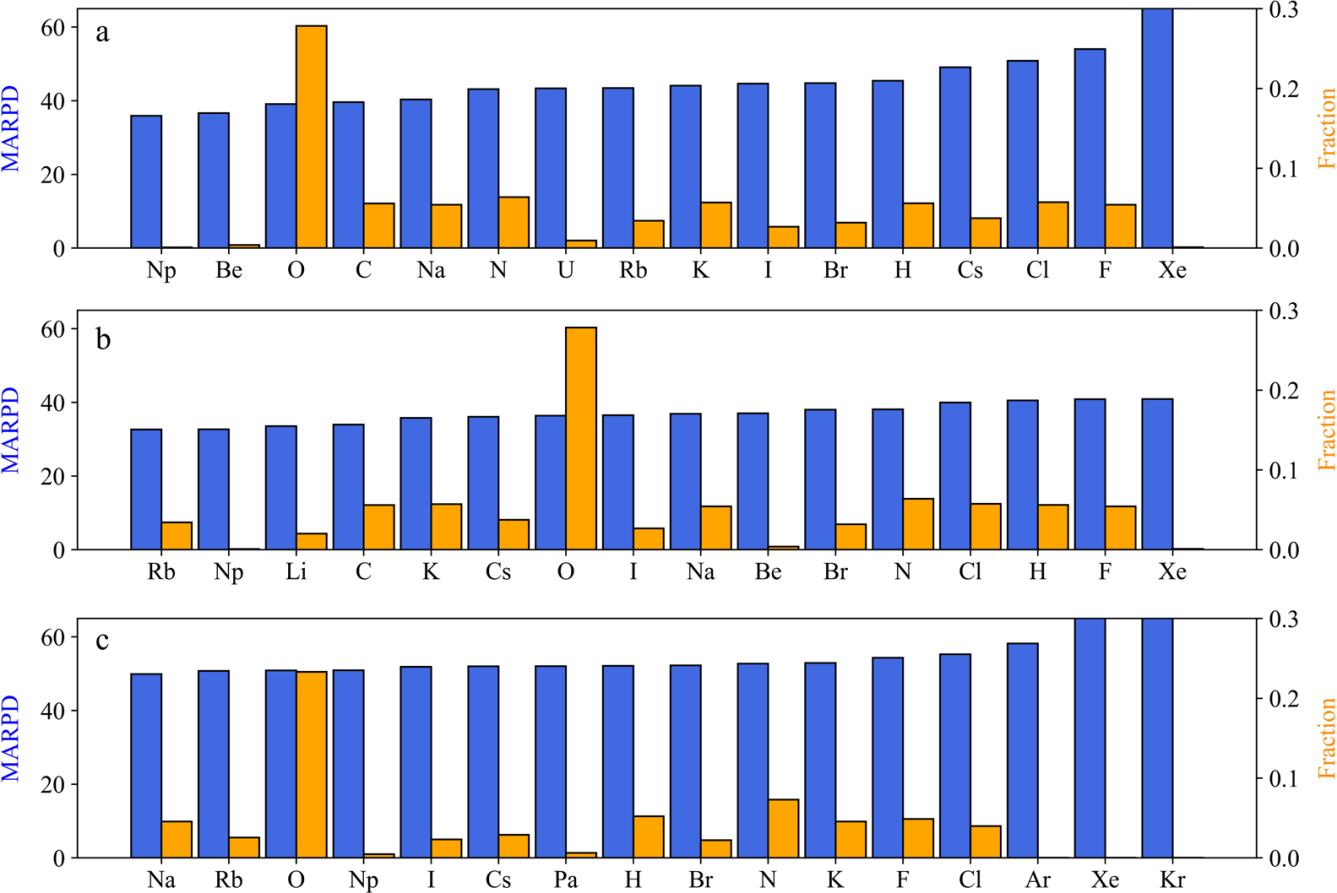
MARPD values (left axis, blue bars) and the fraction of elements (right axis, orange bars) counted from the test set crystals. Results are shown for the M2SM (a), EEM (b) trained with the M2SD, and NeN model (c) trained with the MP2022 data set.

We previously mentioned that element embeddings significantly impact our predictions; thus, we analyze their contribution to prediction quality. Figure 7 presents two key metrics: the eDOS MARPD values and the fraction of crystals containing each selected element. Most crystals contain multiple elements, so the sum of all fractional values can exceed one. Additionally, not all elements are present in every crystal, leading to smaller total values on the fraction axis. Thus, these values should be interpreted relative to each other. A close inspection of the fraction values aims to identify the underlying reasons for high error: (1) if a high error corresponds to a small fraction, it may indicate a small number of structures containing such elements in the training data set, leading to poor prediction accuracy for these structures. Conversely, (2) large MARPDs alongside high fractions may indicate other issues, such as model inaccuracies or drawbacks in reference data.

The disparities in MARPD values across elements highlight the complexities in predicting the electronic properties of specific crystals. Figure 7a (M2SM) shows that crystals with alkali metals (Cs, Rb, K, Na), halogens (Cl, Br, I, F, H), and elements like Xe, C, N, and O exhibit the highest errors. Crystals with Xe have the lowest fraction, indicating they were underrepresented in the training set. On the other hand, halogens and oxygen-containing crystals, despite having high fraction values, also show high MARPD. This implies that the model failed to capture the structure–property relationship of these crystals, indicating potential limitations in the model’s predictive capabilities.

Similarly, the EEM results (Figure 7b) show a similar pattern where alkali metals (Na, Cs, Li, Rb) and halogens (Cl, Br, I, F, H) exhibit the highest prediction errors. Crystals containing N, C, and O also pose significant challenges for accurate eDOS prediction. A similar qualitative trend is observed in the NeN model of the MP2022 data sets (Figure 7c). The primary difference is the emergence of a third group of elements – Kr, Ar, and Xe–which show high errors and low fractions in the test set crystals.

Based on these observations, it can be noted that quantum chemical calculations involving compounds with single valence electron alkali metals, highly electronegative halogens, closed-shell inert gases, as well as those containing N, C, and O, present significant challenges due to strong electron correlation effects. This can result in higher absolute eDOS values. Table S1 in the Supporting Information provides evidence, showing the averaged maximum spectra values for crystals containing certain elements. Crystals with oxygen have eDOS values 1.5–2 times higher than those without oxygen. The total difference across selected elements can be up to 3×. Consequently, smaller spectra values result in smaller errors, while higher values lead to larger errors. Nonetheless, our approach can offer preliminary insights into the electronic characteristics of different crystals. Depending on the objectives, the initially estimated Fermi level energies and band gap values can serve as guides for more resource-intensive quantum chemical calculations.

The EEM workflow is designed to be simpler, faster, and scalable without compromising predictive accuracy. Besides, it is flexible, allowing for the inclusion of extra input parameters. For example, both the M2SM and APET models predict standardized spectra over a fixed range of energies originating from the Fermi level; they do not provide information about the Fermi level and do not take an array of energies as input. In contrast, our models trained on the MP2022 data set include the energy range as an input, enabling the prediction of the Fermi level together with band gap values. Architectural changes for this fitting require only one more input branch and two additional output neurons over the LSTM layer, with these modifications being adaptable to the task without significantly impacting computational time and resources. Particularly in this work, our approach is advantageous because it aligns with the fitting of quantum-level computed data, where the Fermi level is an output of quantum computations, like eDOS spectra.

In the context of computational resources, our approach achieves a notable reduction in computational costs compared to competing models. Specifically, it requires about 20 ms for single crystal predictions on a single Nvidia V100 GPU, as opposed to the seconds and minutes required by GNNs.^11,30^ This underscores its practicality and scalability for handling large-scale screening tasks in materials science and computational chemistry research.

## 5 Conclusions

We introduce a deep machine learning model for predicting the electronic properties of crystal compounds. By encoding crystals through the pair distribution function and the atomic types embedding layer, we capture the chemical information on crystals, which is then stored in a machine learning database. Applied to the original Mat2Spec data set, our model demonstrates competitive performance with the original Mat2Spec model. In the Mat2Spec data set example, we show the pivotal role of element embeddings in our approach. Moreover, trained on the MP2022 data set, our workflow can predict additional scalar quantities. Predicting Fermi level energy, band gap and normalized eDOS values within the same model achieves MAE values comparable to those of deep graph neural networks. In particular, the band gap MAE of 0.196 eV is the best result among all known machine learning models used on the materials considered. The predicted eDOS spectra match the references qualitatively. In essence, our study provides a flexible and efficient machine learning framework. It can serve as a springboard for future research endeavors but would already be useful for the screening of chemical compounds and materials.

## Data Availability

All the data used in this paper are publicly available and can be accessed from various sources. The structure files for the MP data set are accessible at https://matbench.materialsproject.org/. The source code used in this study is available at https://github.com/dmamur/struct2prop. Detailed Python notebooks for replicating all calculations and accessing trained model checkpoints are provided on the corresponding GitHub page.

## Supporting Information Available

Additional details of data collection and preprocessing, explanation of PDF inputs to the LSTM module, illustrations of various model architectures, comparisons of predicted and reference eDOS spectra across different models, and averaged maximum spectra values for crystals containing selected elements (PDF)
