# Supplementary material for: Structure to Property: Chemical Element Embeddings for Predicting Electronic Properties of Crystals
Source: J Chem Inf Model. 2024 Jul 15;64(15):5762–70. doi: 10.1021/acs.jcim.3c01990 (PMC11323004; doi:10.1021/acs.jcim.3c01990)
Supplement: Supplementary file 1 — ci3c01990_si_001.pdf [file ci3c01990_si_001.pdf]

Supporting Information:

Structure to Property: Chemical Element Embeddings for Predicting Electronic Properties of Crystals

Shokirbek Shermukhamedov*[,1], Dilorom Mamurjonova[2], Thana Maihom[3,4], and Michael Probst[1,3]

[1]Institute of Ion Physics and Applied Physics, University of Innsbruck, 6020 Innsbruck, Austria
[2]Department of Inorganic Chemistry, Tashkent Chemical Technological Institute, 100011 Tashkent, Uzbekistan [3]School of Molecular Science and Engineering, Vidyasirimedhi Institute of Science and Technology, 21201 Rayong, Thailand
[4]Division of Chemistry, Department of Physical and Material Sciences, Faculty of Liberal Arts and Science, Kasetsart University, Kamphaeng Saen Campus, 73140 Nakhon Pathom, Thailand.

* Corresponding Author
E-mail: shokirbek.shermukhamedov@uibk.ac.at

Section 1

We collected a total of 126,334 compounds from the Materials Project database, out of which 88,058 contain eDOS spectra. However, not all of these eDOS spectra were calclated with the same quantum chemical methods; specifically, they were derived using various GGA and GGA+U functionals. For the of training our model we exclusively employed the 69,178 GGA-calculated eDOS spectra.

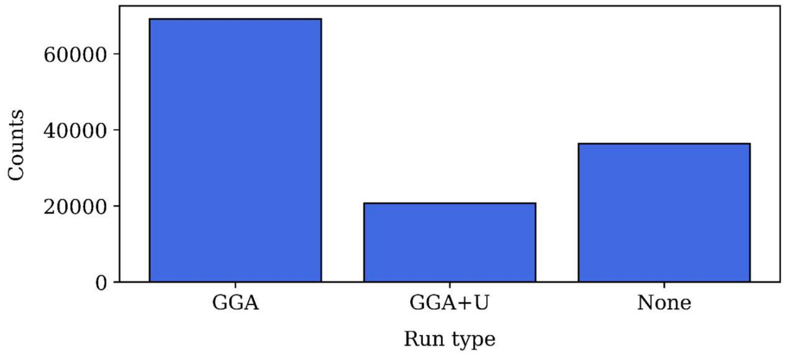

Figure S1. Distribution of density functionals used for the calculation of the eDOS spectra of crystals. "None" indicates that no eDOS calculations were performed for these structures.

In the dataset comprising 69,178 digitized spectra, uniformity in terms of length and energy resolution (dE) is not always observed. Arrays predominantly consisted of 301, 601, and 2001 datapoints, as depicted in the subplot of Figure S4. For our analysis, only spectra with 2001 datapoints were utilized, as indicated by the red bars in Figure S2.

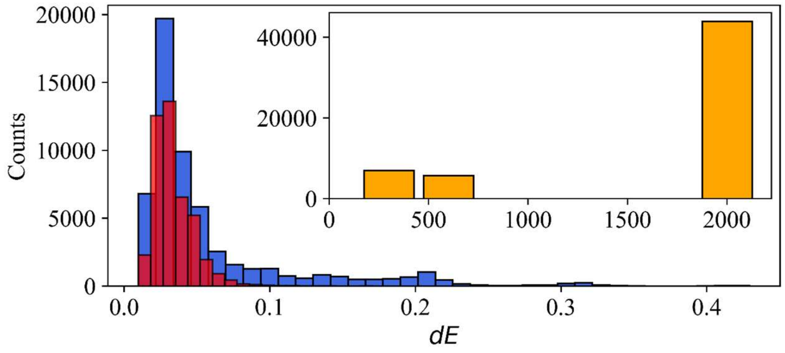

Figure S2. Distribution of eDOS data based on the data length and energy resolution.

Section 2.

The pair distribution function (PDF) of an individual atom is input to the LSTM module. It is defined as the probability of locating an atom at a given distance (r) from a selected atom, compared to the unbiased probability:

$$PDF(r) = \frac{V}{N(N-1)} \frac{dn_r}{4\pi r^2 dr}$$

where,

- $PDF(r)$ is the pair distribution function.
- V is the volume of the system.
- N is the total number of particles.
- dn_r is the probability of finding two particles in a shell of thickness dr at a distance between r and $r + dr$.
- $4\pi r^2$ is the surface area of the shell.

The PDF was restricted to 10Å and dr was selected as 0.1Å. Overall each PDF vector length was 100.

Section 3

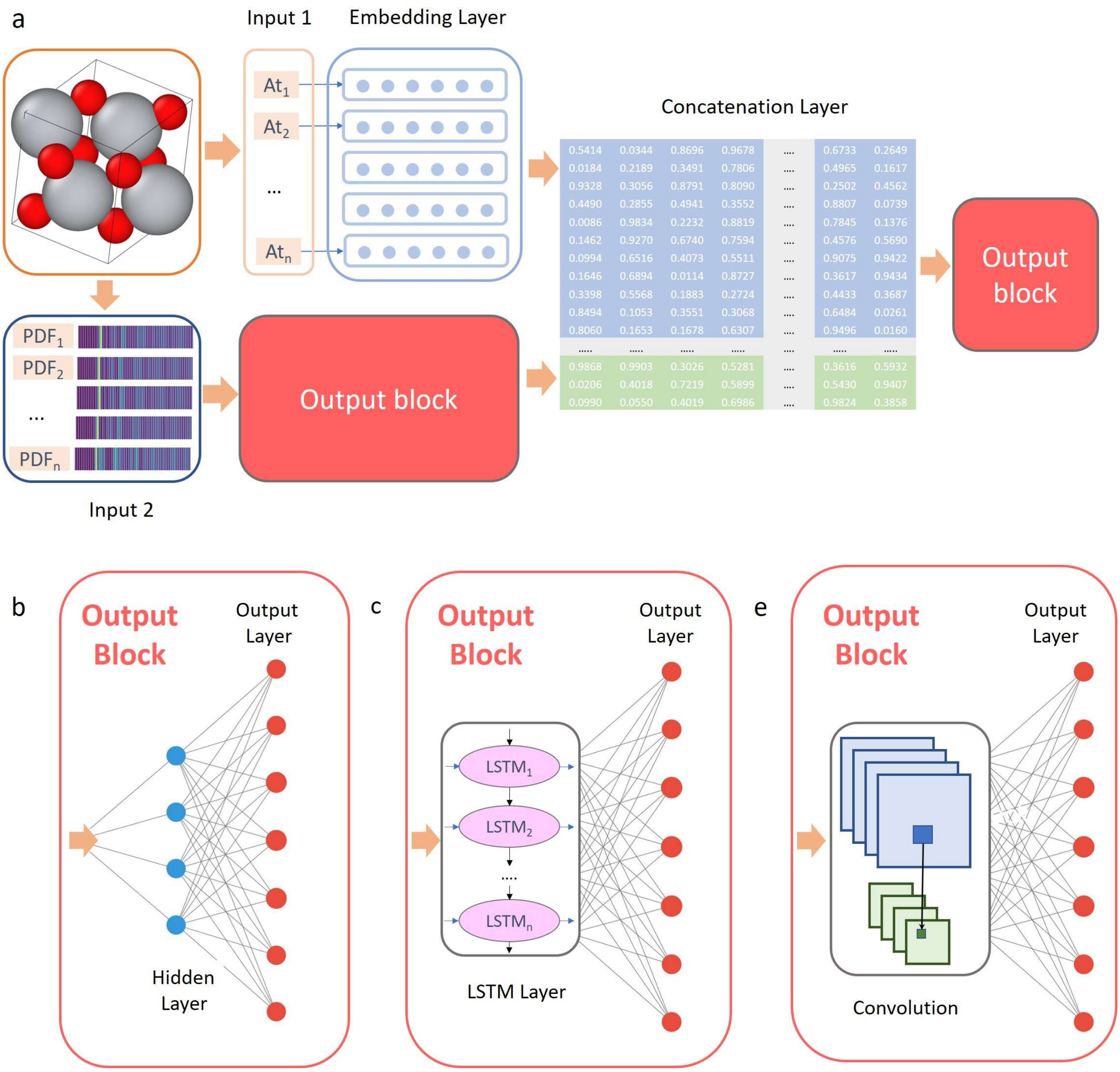

Figure S3. Flexible model architecture (a) and possible layer types: (b) dense layers; (c) LSTM layers like in the original model; (d) Convolution neural networks combined with output dense layers.

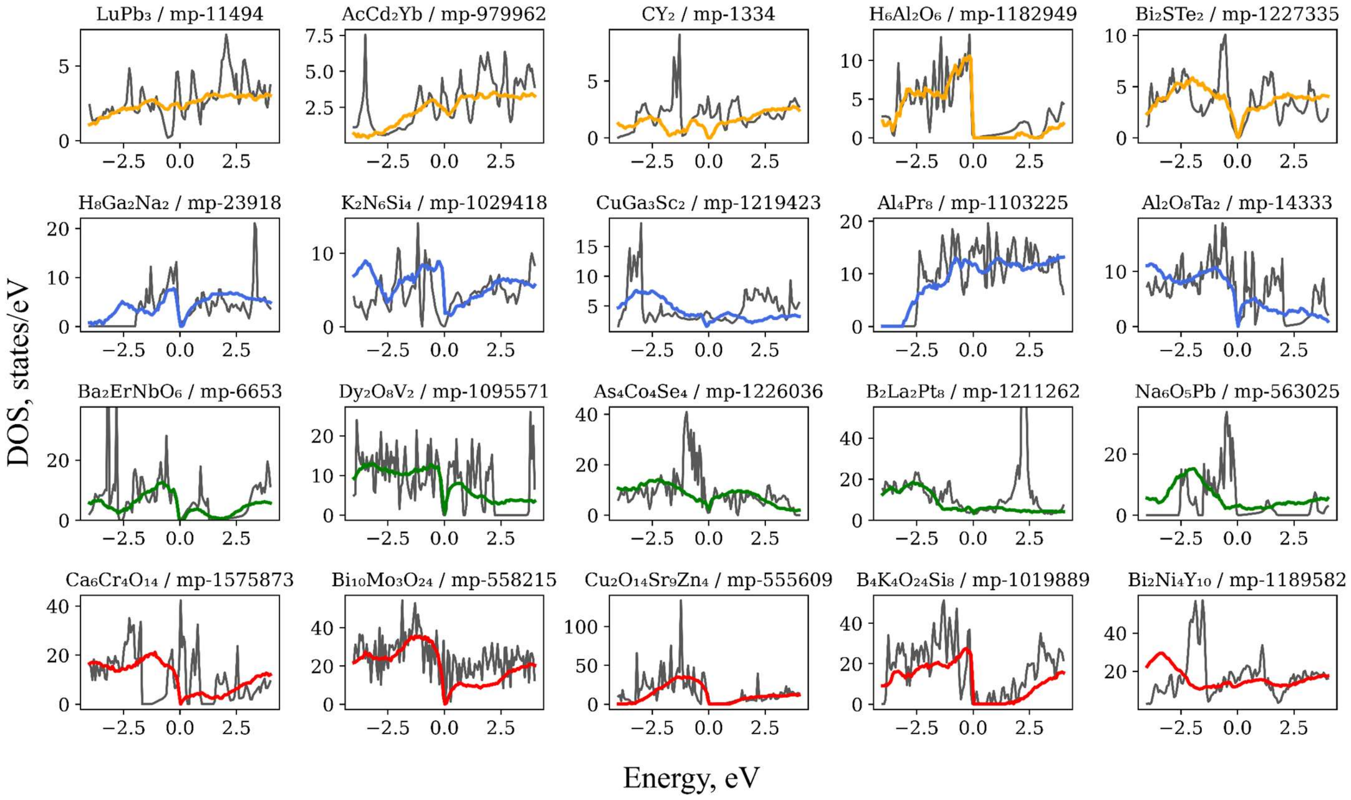

Figure S4. eDOS examples, taken from the test set of the Mat2Spec dataset (black) and predicted spectra (colored) trained without an element embedding layer. The examples are five representative materials chosen from each quartile from low MAE (top) to high MAE (bottom) of our model predictions, represented by line colors transitioning from orange to red. The chemical sum formula and ID number of each material are shown above each subplot.

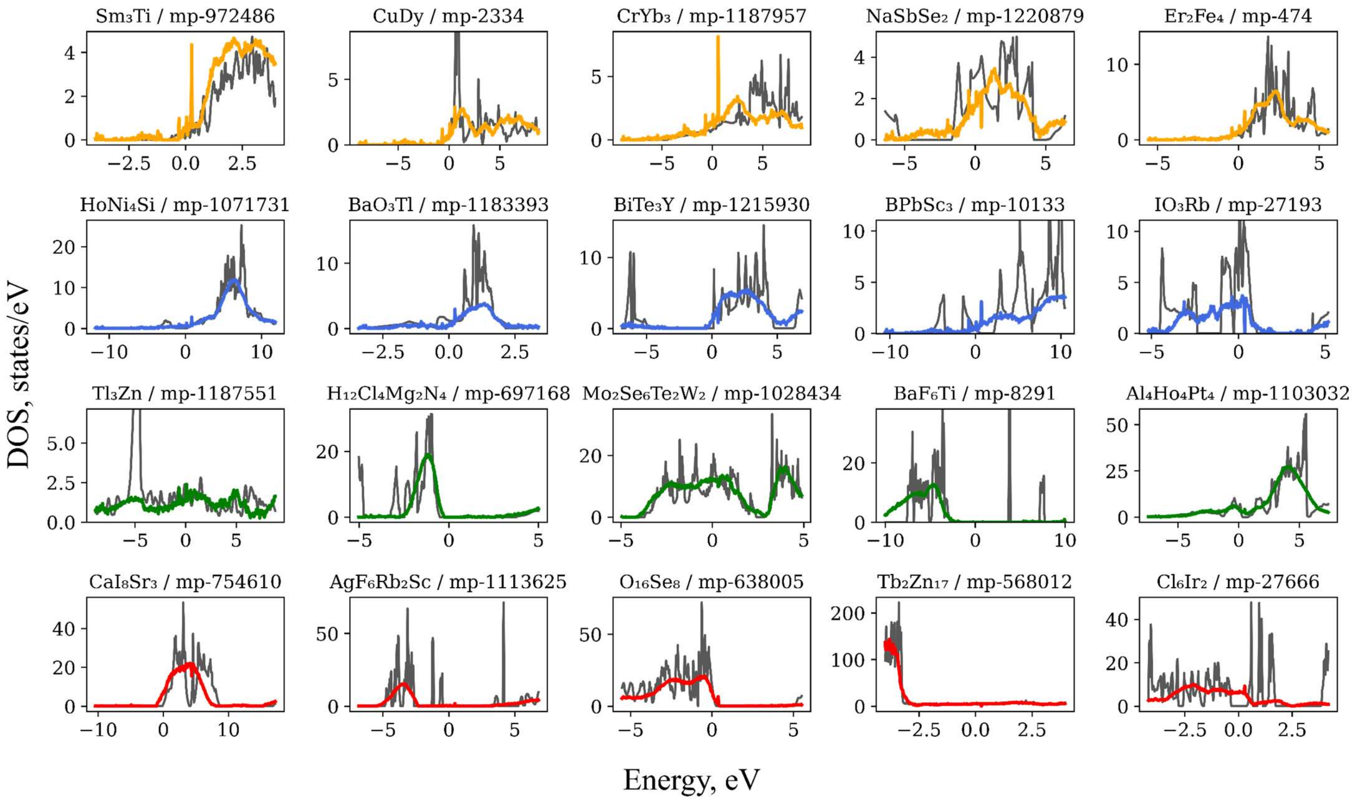

Figure S5. eDOS examples, taken from the test set of the MinMax normalized MP2022 dataset (black), and predicted spectra (colored). The examples show five representative materials chosen from each quartile from low MAE (top) to high MAE (bottom) of our model predictions, represented by line colors transitioning from orange to red. The chemical sum formula and ID number of each material are shown above each subplot.

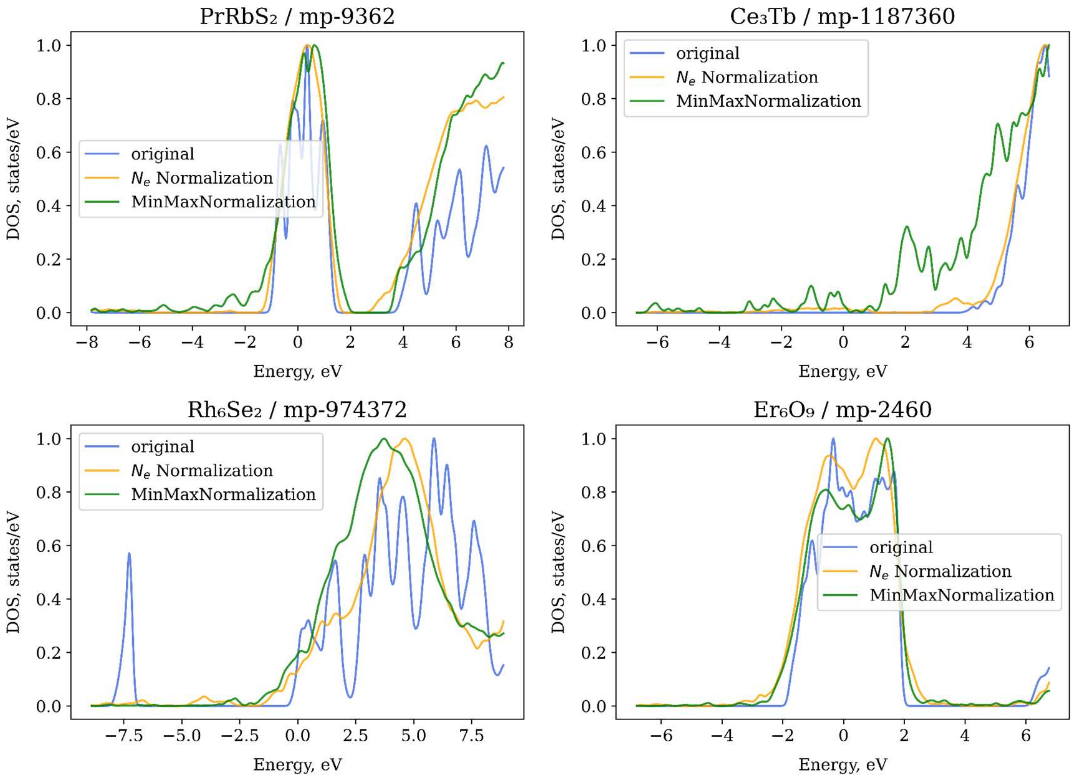

Figure S6. Four representative examples from the model trained on MinMax and N_e-normalized MP2022 dataset. The data sets were normalized to the maximal values to ensure accurate comparisons.

Table S1. Averaged maximum values of eDOS spectra for selected elements in the test sets. The sign "+" in the name of the dataset indicates the presence of crystals containing the element in the first column, and "-" – all crystals without this element.

Elements	M2SD+	M2SD-	MP2022+	MP2022-
**O**	57.49	38.21	120.53	58.87
**N**	52.13	42.99	60.37	74.25
**C**	51.19	43.13	49.58	74.60
**H**	58.75	42.67	70.93	73.37
**F**	83.14	41.30	96.22	72.06
**Cl**	68.33	42.07	94.35	72.36
**I**	60.76	43.11	97.88	72.66
**Br**	63.99	42.91	80.83	73.07
**Na**	52.99	43.03	80.68	72.88
**K**	60.56	42.55	97.82	72.07
**Rb**	57.43	43.09	81.26	73.03
**Cs**	66.75	42.67	96.70	72.54
**Be**	137.76	43.21	84.24	73.11
**Ta**	33.34	43.85	69.73	73.33
**Fe**	38.77	43.65	35.32	74.34
**W**	36.74	43.76	30.37	74.05
**Ti**	36.90	43.81	321.77	63.10
